# Predicting the Moisture Ratio of a Hami Melon Drying Process Using Image Processing Technology

**DOI:** 10.3390/foods12030672

**Published:** 2023-02-03

**Authors:** Guanyu Zhu, G.S.V. Raghavan, Zhenfeng Li

**Affiliations:** 1 Jiangsu Key Laboratory of Advanced Food Manufacturing Equipment and Technology, School of Mechanical Engineering, Jiangnan University, Wuxi 214122, China; 2 Department of Bioresource Engineering, McGill University, 21111 Lakeshore Road, Sainte-Anne-de-Bellevue, QC H9X 3V9, Canada

**Keywords:** image processing, Hami melon drying, moisture ratio model, adjustable-power microwave drying system, shrinkage, maximum likelihood

## Abstract

For food drying, moisture content and shrinkage are vital in the drying process. This paper is concerned with the moisture ratio modeling and prediction issues of the Hami melon drying process. First, an experimental system was developed; it included an adjustable-power microwave drying unit and an image-processing unit. The moisture contents and the areas of Hami melon slices at different times were sampled in real time. Then, the expression of the moisture ratio with regard to shrinkage was derived by using the Weierstrass approximation theorem. A maximum likelihood fitness function-based population evolution (MLFF-PE) algorithm was then put forward to fit the moisture ratio model and predict the moisture ratio. The results showed that the proposed MLFF-PE algorithm was effective at fitting and predicting the moisture ratio model of the drying process of Hami melon slices.

## 1. Introduction

During food-drying processes, a variety of physical and chemical changes occur inside foods, which affect the quality of dried products [1,2,3]. The changes in the moisture content in high-moisture foods, such as Hami melons, generally result in shape changes [4,5,6]. This kind of shape change is expressed as shrinkage [7]. For modeling food-drying processes, moisture content and shrinkage are two important indicators that reveal the drying schedule [8,9,10], and they play significant roles in many aspects [11,12,13]. For instance, if the relationship between the moisture content and the shrinkage is built, we will be able to obtain the moisture content in real time by detecting the shrinkage, knowing the end point of the drying process, or predicting the drying process. That is to say, according to the modeling results, we can decide when to finish the drying process or design microwave power-adjusting strategies to obtain dried products with better quality. In recent decades, many researchers have studied different mathematical models of moisture content and shrinkage during food-drying processes [14,15,16]. Yadollahinia et al. studied the drying characteristics of potato slices and pointed out that the dimensionless area changes of potato slices decreased linearly as the dimensionless moisture content decreased [17]. Afonso et al. employed the three-order polynomial to describe the relationship between the physical characteristics of coffee berries, such as the volume shrinkage and moisture content measured in drying experiments [18]. During the drying process of pineapple slices, a linear function containing exponential constants was used to express the relationship between the shrinkage and moisture content [19]. In [17,18,19], the models were shrinkage versus moisture content. In this paper, we established the mathematical model of the moisture ratio with regard to the shrinkage during the Hami melon drying process as the *n*-order polynomial in line with Weierstrass Approximation theorem, and developed a new algorithm to realize model fitting.

In recent years, image processing has been widely applied in various drying systems [20,21,22]. By building the relationships between the visual appearances of foods, such as the color, size, and shape, and the easily measured quality attributes of foods, such as the moisture content, density, and porosity at different stages of the drying processes, image processing has been used to evaluate the qualities of dried foods at specific times in drying processes [23,24]. Madiouli et al. studied the shrinkage kinetics of bananas and observed that banana slices showed ideal shrinkage at the beginning of drying, but stopped shrinkage with low moisture content [25]. Huang et al. used hyperspectral imaging technology to capture the images of soybeans at different stages in microwave drying. Using those images, they studied the relationship between the color changes of soybeans and the moisture content and concluded that there was a correlation between the hyperspectral reflectance, entropy, and moisture content [26]. In the microwave vacuum drying process of carrots, Nahimana and Zhang exploited the image processing software called ImageJ to monitor the shrinkage and color changes of carrots [27]. Nevertheless, the image processing technology used in most research studies on drying, such as in [25,26,27], is offline, which means that the products must be taken out to take photos and, thus, the drying processes must be interrupted. To overcome this shortcoming, this paper developed an image processing-based microwave drying system to study the changes in the moisture ratio of Hami melons by detecting shrinkage during the drying process.

In the area of modeling and estimation, the maximum likelihood method is usually utilized because it has good statistical properties and can be applied to linear and nonlinear models [28,29,30]. The core idea of the maximum likelihood method is that the estimated values are obtained by maximizing the probability of the occurrence of the experimental data [31,32,33]. Xie et al. adopted the maximum likelihood method to recover the parameters of Bernoulli autoregressive models [34]. Wu et al. developed a new method for the joint amplitude and noise variance estimation of a single sinusoid on the basis of the maximum likelihood method [35]. Çayır and Candan investigated the autoregressive model parameter estimation issues by using the maximum likelihood method [36]. By taking advantage of the maximum likelihood method, this paper derived the maximum likelihood fitness function and studied a novel algorithm to solve the moisture ratio modeling problem of the Hami melon drying process.

The main contributions of this paper are as follows.

1. An experimental system that included an adjustable-power microwave drying unit and an image-processing unit was developed, and the moisture content and the area of samples at different times during the Hami melon drying process were collected.
2. The representation of the moisture ratio with regard to the shrinkage of the drying process of Hami melon slices was assumed by means of the Weierstrass approximation theorem.
3. By deducing the maximum likelihood fitness function, a maximum likelihood fitness function-based population evolution (MLFF-PE) algorithm was presented to fit the moisture ratio model and predict the moisture ratio changes in the drying process of Hami melon slices. The results showed that the estimated moisture ratio model given by the MLFF-PE algorithm performed well in the moisture ratio model’s fitting and the moisture ratio prediction of the Hami melon drying process.

This paper is organized as follows. Section 2 presents an experimental system, including an adjustable-power microwave drying unit and an image-processing unit, and introduces the experimental procedure of the Hami melon drying process. Section 3 presents the mathematical model of the moisture ratio versus the shrinkage. In Section 4, the MLFF-PE algorithm is presented to fit the moisture ratio model. Section 5 provides the results of model fitting and prediction. Finally, the conclusions and future work are summarized in Section 6.

## 2. Materials and Methods

### 2.1. Materials

Fresh Hami melons (*Cucumis melo* var. *saccharinus*) were purchased at a local market in Wuxi, China. Undamaged Hami melons with moderate maturity and clear skin lines were selected and stored at 5 °C and then placed at room temperature at 20 °C for 30 min before drying. They were washed, peeled, and cut into 6 mm slices using a mechanical cutter. The slices were then cut into 25 mm diameter cylindrical pieces with a cutting tool. The initial moisture contents of fresh Hami melon samples were measured and obtained, i.e., 9.87 g/g on a dry basis by drying with hot air at 105 °C for 24 h, which was adequate to obtain the constant mass of the slices.

### 2.2. Microwave Drying System Based on Image Processing

**Figure 1:**
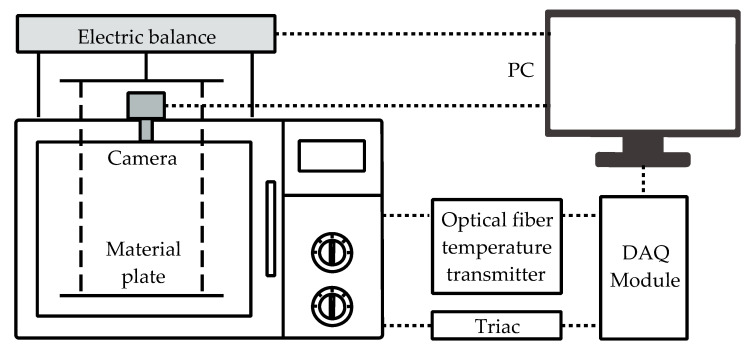
The schematic of the system.

The developed experiment system consisted of an image-processing unit and a microwave drying unit. The schematic of the system is shown in Figure 1.

In the image-processing part, 3 LED light strips were applied as light sources, which were installed on the door of the microwave oven. A hole with a diameter of 6 mm was drilled at the top of the microwave oven for imaging and an industrial camera (SKT-SL1200C-123A, Chengyishun Tech. Co., Ltd., Shenzhen, China) was installed above the hole. The collected images were transmitted to the PC via a USB cable. As for the microwave-drying part, a 700 W microwave oven (EM7KCGW3-NR, Midea Co., Ltd., Guangzhou, China) was utilized for drying, where the original circuit was modified to make the microwave power continuously adjustable with the help of a Triac and a data acquisition (DAQ) board (USB 6008, National Instruments Corp., Austin, TX, USA). The sample holding plate was supported by an electric balance above the microwave oven cavity through 4 Teflon sticks for mass measurements. The electronic scale was able to read the mass information in real time and transfer the data to the PC through the RS232 to USB cable. The precision of the electronic balance was 0.01 g. An optical fiber sensor (HQFTS-PAA0A-0300, Xian Heqi Photoelectric Tech. Co., Ltd., Xi’an, China) was inserted into the core of one of the samples to take the temperature [37]. The collected optical signal representing the temperature was converted into a DC voltage signal through the temperature transmitter and was recognized by the PC via the DAQ module. The temperature error was ±1 °C.

### 2.3. Experimental Details

After several pre-experiments, considering the drying time and quality after drying, 60 °C was selected as the microwave drying temperature of the Hami melon. The temperature of the material core was measured in real-time by the optical fiber, and the constant drying temperature of 60 °C was achieved by continuously adjusting the power under a PID control strategy [38]. A fan installed on the side wall of the microwave oven drew out the hot air in the oven to achieve airflow. Mass was measured during the drying process and the image-processing algorithm was performed every 30 s, followed by data recording. Drying was stopped when the dry basis moisture content reached 0.176 g/g. The total drying time was approximately 41 min. The experiments were performed in triplicates.

### 2.4. Image Processing Algorithm

**Figure 2:**
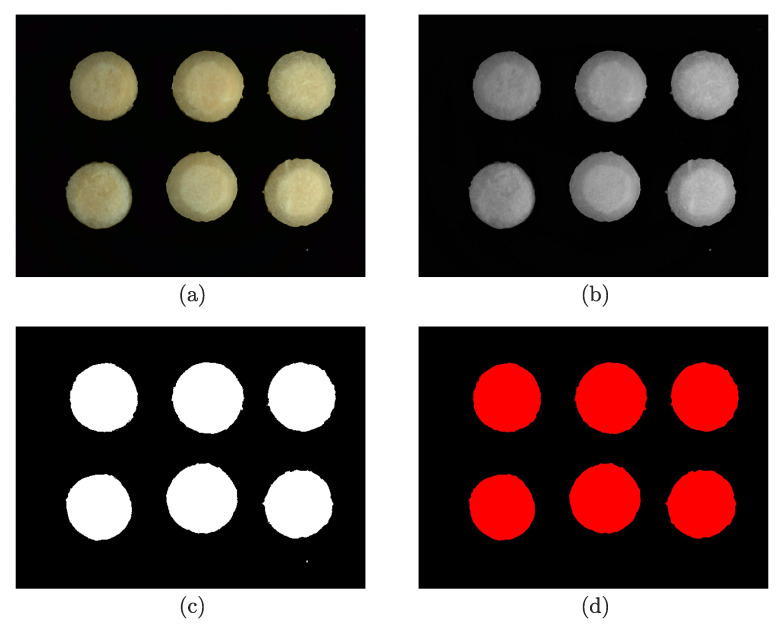
The image processing steps: (**a**) original RGB image before processing; (**b**) grayscale image; (**c**) binary image; (**d**) image after the operation of filling holes and filtering particles.

An image-processing algorithm was developed to monitor the shrinkage of the material. The software, Vision and Motion Module, based on LabVIEW (Version 16.0; National Instruments Corp., Austin, TX, USA), was utilized to implement the algorithm. The major steps of image processing are summarized below and a typical example of the steps is illustrated in Figure 2.

Step 1: Capture and read the 24-bit RGB image—see (a) of Figure 2.

Step 2: Convert the RGB image into an 8-bit grayscale image by averaging the 3 color components of the RGB image—see (b) of Figure 2.

Step 3: Transform the grayscale image into a binary image by computing the optimal threshold value using the clustering method—see (c) of Figure 2.

Step 4: Fill in the holes found in each particle to remove the image noise inside the samples.

Step 5: Filter and retain the top 6 image particles in the area to eliminate the image noise outside the samples—see (d) of Figure 2.

Step 6: Extract and sum the pixel counts of each filtered particle.

## 3. Mathematical Model

In this section, the definitions of the moisture ratio and the shrinkage are given and the relationship between the moisture ratio and the shrinkage is provided.

During the drying process, the moisture content and the moisture ratio of the samples were, respectively, calculated by the following equations:

$$\begin{array}{ccc} MC(t) & = & \frac{m\left(t\right)-m_{d}}{m_{d}},\\ MR(t) & = & \frac{MC(t)}{MC(0)}, \end{array}$$

where *t* represents the drying time (min), m(t) stands for the real mass of the samples at time *t* (g), $m_{d}$ stands for the mass of dry matter of the samples (g), MC(t) denotes the moisture content at time *t* (g/g), MC(0) denotes the initial moisture content at time t=0 (g/g), and MR(t) symbolizes the moisture ratio at time *t* (dimensionless).

The shrinkage of the samples, which was regularly quantified as the area ratio, was computed by the following equation:

$$S\left(t\right)=\frac{A(t)}{A(0)},$$

where A(t) stands for the area at time *t*, which is expressed by the number of pixels of the samples (px), A(0) stands for the initial area of the samples (px), and S(t) symbolizes the shrinkage of the samples at time *t* (dimensionless).

To study the relationship between the moisture ratio and the shrinkage, the moisture ratio was described as a function of the shrinkage:

(1) $$\begin{array}{ccc} \hat{MR}\left(t\right) & = & f(S(t)), \end{array}$$

(2) $$\begin{array}{ccc} MR(t) & = & \hat{MR}\left(t\right)+v\left(t\right), \end{array}$$

where S(t) is the shrinkage of the samples, $\hat{MR}\left(t\right)$ is the function value, MR(t) is the experimental moisture ratio, and v(t) is the measurement error; it is supposed to be an independent zero-mean white Gaussian noise with variance $\sigma^{2}$.

Theorem 1 (**Weierstrass approximation theorem).** *Let f(x) be continuous on an interval C. Then for any ϵ>0, there exists a polynomial p(x), such that*

|p(x)−f(x)|<ϵ,∀x∈C.

According to Theorem 1, it can be deduced that any continuous function f(x) can be approximated arbitrarily well by means of a polynomial p(x) with the required accuracy. Therefore, we utilized the *n*-order polynomial p(·) to replace the function f(·) in (1) and rewrite (1) and (2) as

(3) $$\begin{array}{cc} \hat{MR}\left(t\right) & =p(S(t))\\ & =a_{0}+a_{1}S\left(t\right)+a_{2}S^{2}\left(t\right)+\cdots +a_{n}S^{n}\left(t\right), \end{array}$$

(4) $$\begin{array}{ccc} MR(t) & = & \hat{MR}\left(t\right)+v\left(t\right). \end{array}$$

The parameter vector α and the information vector φ(t) are defined as

$$\begin{array}{ccc} \alpha & = & {\left[a_{0},a_{1},a_{2},\cdots ,a_{n}\right]}^{T}\in R^{Q},\;Q=n+1,\\ \varphi (t) & = & {\left[1,S\left(t\right),S^{2}\left(t\right),\cdots ,S^{n}\left(t\right)\right]}^{T}\in R^{Q}. \end{array}$$

Inserting (4) into (3), we could describe the relationship between the moisture ratio and the shrinkage by

(5) $$\begin{array}{ccc} MR(t) & = & a_{0}+a_{1}S\left(t\right)+a_{2}S^{2}\left(t\right)+\cdots +a_{n}S^{n}\left(t\right)+v\left(t\right)\\ & = & \left[1,S\left(t\right),S^{2}\left(t\right),\cdots ,S^{n}\left(t\right)\right]\left[\begin{array}{c} a_{0}\\ a_{1}\\ a_{2}\\ \vdots \\ a_{n} \end{array}\right]+v\left(t\right)\\ & = & \varphi^{T}\left(t\right)\alpha +v\left(t\right). \end{array}$$

The objective of this paper was to propose a novel parameter estimation algorithm to identify the model in (5) from the collected discrete experimental data $S(t_{k})$ and $MR(t_{k})$ at the discrete sampling time $t=t_{k}$ (k=1, 2, ⋯, *D*). Moreover, in this paper, we adopted the uniform sampling method with the sampling period ΔT. Thus, the experimental data $S(t_{k})$ and $MR(t_{k})$ could be represented as S(kΔT) and MR(kΔT) or S(k) and MR(k) for short.

## 4. MLFF-PE Method

In this section, we developed the maximum likelihood fitness function-based population evolution (MLFF-PE) algorithm to identify the parameter vector α of the model in (5).

### 4.1. Population Initialization

The MLFF-PE algorithm began with the generation of an initial population. In consideration of the *Q*-dimensional parameter vector to be estimated α in (5), the population size was supposed to be P∈R and the initial population was defined as

(6) $${\hat{\Lambda }}^{0}={\left[{\hat{\alpha }}_{1}^{0},{\hat{\alpha }}_{2}^{0},\cdots ,{\hat{\alpha }}_{P}^{0}\right]}^{T}\in R^{P\times Q},$$

which consisted of *P* initial individuals (i.e., the parameter vectors to be estimated) from ${\hat{\alpha }}_{1}^{0}$ to ${\hat{\alpha }}_{P}^{0}$. In the initial population ${\hat{\Lambda }}^{0}$, the *p*th initial individual was

(7) $${\hat{\alpha }}_{p}^{0}={\left[{\hat{\alpha }}_{p,1}^{0},{\hat{\alpha }}_{p,2}^{0},\cdots ,{\hat{\alpha }}_{p,Q}^{0}\right]}^{T}\in R^{Q},\;p=1,2,\cdots ,P,$$

where *p* is the index for the individuals. Each element in the *p*th initial individual ${\hat{\alpha }}_{p}^{0}$ was randomly generated as follows:

(8) $${\hat{\alpha }}_{p,q}^{0}=\mathrm{rand}\left(0,1\right),\;q=1,2,\cdots ,Q,$$

where *q* is the index for the elements in the *p*th individual and rand(0,1) is a uniformly distributed stochastic number between 0 and 1. Thus, the initialization process of the population was completed.

Because the population and the individuals were changed with the different evolution generations, we defined the population ${\hat{\Lambda }}^{gen}$ and the individual ${\hat{\alpha }}_{p}^{gen}$ at the generation $gen\in [0,gen_{\max}-1]$ as

(9) $$\begin{array}{ccc} {\hat{\Lambda }}^{gen} & = & {\left[{\hat{\alpha }}_{1}^{gen},{\hat{\alpha }}_{2}^{gen},\cdots ,{\hat{\alpha }}_{P}^{gen}\right]}^{T}, \end{array}$$

(10) $$\begin{array}{ccc} {\hat{\alpha }}_{p}^{gen} & = & {\left[{\hat{\alpha }}_{p,1}^{gen},{\hat{\alpha }}_{p,2}^{gen},\cdots ,{\hat{\alpha }}_{p,Q}^{gen}\right]}^{T}. \end{array}$$

Each individual ${\hat{\alpha }}_{p}^{gen}$ in the population ${\hat{\Lambda }}^{gen}$ was a possible estimate of the parameter vector α.

### 4.2. Mutation Process

After the initialization process of the population, the mutation process was realized to produce the mutant vector for each individual ${\hat{\alpha }}_{p}^{gen}$.

The mutant vector ${\hat{\beta }}_{p}^{gen}$ was defined as

(11) $${\hat{\beta }}_{p}^{gen}={\left[{\hat{\beta }}_{p,1}^{gen},{\hat{\beta }}_{p,2}^{gen},\cdots ,{\hat{\beta }}_{p,Q}^{gen}\right]}^{T}\in R^{Q}.$$

At this stage, the mutant vector ${\hat{\beta }}_{p}^{gen+1}$ was produced by adding the vectorial difference between the second and third individuals to the first individual:

(12) $${\hat{\beta }}_{p}^{gen+1}={\hat{\alpha }}_{r_{1}}^{gen}+F\cdot \left({\hat{\alpha }}_{r_{2}}^{gen}-{\hat{\alpha }}_{r_{3}}^{gen}\right),\;r_{1},r_{2},r_{3}\in \left[1,P\right],$$

where F∈R is a positive constant called the scaling factor, which controls the magnitude of the vectorial difference ${\hat{\alpha }}_{r_{2}}^{gen}-{\hat{\alpha }}_{r_{3}}^{gen}$, and $r_{1}$, $r_{2}$, and $r_{3}$ are integers stochastically selected from the set {1, 2, ⋯, P}, and those three integers ($r_{1}$, $r_{2}$, and $r_{3}$) are not equal to each other or to the index *p*.

### 4.3. Crossover Process

After producing the mutant vector ${\hat{\beta }}_{p}^{gen+1}$ during the mutation process, the crossover process was implemented to enhance population diversity. The crossover vector ${\hat{\lambda }}_{p}^{gen}$ was defined as

(13) $${\hat{\lambda }}_{p}^{gen}={\left[{\hat{\lambda }}_{p,1}^{gen},{\hat{\lambda }}_{p,2}^{gen},\cdots ,{\hat{\lambda }}_{p,Q}^{gen}\right]}^{T}\in R^{Q}.$$

In the crossover process, some elements in the mutant vector ${\hat{\beta }}_{p}^{gen+1}$ and some elements in the individual ${\hat{\alpha }}_{p}^{gen}$ were mixed to construct the crossover vector ${\hat{\lambda }}_{p}^{gen+1}$. The scheme for generating every element in the crossover vector ${\hat{\lambda }}_{p}^{gen+1}$ is shown as follows:

(14) $$\begin{array}{ccc} {\hat{\lambda }}_{p,q}^{gen+1} & = & \left\{\begin{array}{cc} {\hat{\beta }}_{p,q}^{gen+1}, & \mathrm{if}\;\mathrm{rand}\left(0,1\right)<CR\;\mathrm{or}\;q=q_{\mathrm{rand}}\\ {\hat{\alpha }}_{p,q}^{gen}, & \mathrm{if}\;\mathrm{rand}\left(0,1\right)⩾CR\;\mathrm{and}\;q\neq q_{\mathrm{rand}} \end{array}\right., \end{array}$$

where CR∈R is a positive constant called the crossover rate, which controls the probability of preserving elements in the mutant vector ${\hat{\beta }}_{p}^{gen+1}$ or the individual ${\hat{\alpha }}_{p}^{gen}$; $q_{\mathrm{rand}}$ ensures that the crossover vector ${\hat{\lambda }}_{p}^{gen+1}$ obtains at least one element in the mutant vector ${\hat{\beta }}_{p}^{gen+1}$, is an integer stochastically selected from the set {1, 2, ⋯, D}, and is newly produced for each index *p*.

### 4.4. Maximum Likelihood Fitness Function

In this subsection, the maximum likelihood fitness function (MLFF) is deduced for the MLFF-PE algorithm.

For the collected discrete experimental data {S(1), S(2), ⋯, S(D)}, and {MR(1), MR(2), ⋯, MR(D)}, the likelihood function L(MR(1), MR(2), ⋯, MR(D)|S(1), S(2), ⋯, S(D), α) is equal to the joint conditional probability density function of {MR(1), MR(2), ⋯, MR(D)} with the given {S(1), S(2), ⋯, S(D)}, and α:

(15) $$\begin{array}{ccc} & & L(MR(1),MR(2),\cdots ,MR(D)|S(1),S(2),\cdots ,S(D),\alpha )\\ & = & p(MR(1),MR(2),\cdots ,MR(D)|S(1),S(2),\cdots ,S(D),\alpha )\\ & = & p(MR(D)|MR(1),MR(2),\cdots ,MR(D-1),S(1),S(2),\cdots ,S(D),\alpha )\\ & & \times p(MR(D-1)|MR(1),MR(2),\cdots ,MR(D-2),S(1),S(2),\cdots ,S(D-1),\alpha )\\ & & \times \cdots \times p(MR(1)|MR(0),S(1),\alpha )\\ & = & \prod_{k=1}^{D}p\left(\varphi^{T}\left(k\right)\alpha +v\left(k\right)|MR\left(1\right),MR\left(2\right),\cdots ,MR\left(D-1\right),S\left(1\right),S\left(2\right),\cdots ,S\left(k\right),\alpha \right). \end{array}$$

For the reason that v(k) is a white Gaussian noise and is independent of {MR(1), MR(2), ⋯, MR(D−1)}, {S(1), S(2), ⋯, S(k)}, and α, Equation (15) can be rewritten as

(16) $$\begin{array}{ccc} & & L(MR(1),MR(2),\cdots ,MR(D)|S(1),S(2),\cdots ,S(D),\alpha )\\ & = & \prod_{k=1}^{D}p\left(v\left(k\right)\right)+h\\ & = & {\left(2\pi \sigma^{2}\right)}^{-\frac{D}{2}}\exp\left(-\frac{1}{2\sigma^{2}}\sum_{t=1}^{D}v^{2}\left(k\right)\right)+h, \end{array}$$

where *h* denotes a constant. Here, the goal is to maximize the likelihood function L(MR(1), MR(2), ⋯, MR(D)|S(1), S(2), ⋯, S(D), α) in (16), to maximize the joint conditional probability density function of {MR(1), MR(2), ⋯, MR(D)} with the given {S(1), S(2), ⋯, S(D)}, and α. Nevertheless, the above operation is difficult to realize due to the huge computational burden. For the purpose of tackling this issue, we could take the logarithm of the likelihood function L(MR(1), MR(2), ⋯, MR(D)|S(1), S(2), ⋯, S(D), α) in (16), and equivalently maximize the logarithm likelihood function. That logarithm likelihood function is calculated by

(17) $$\begin{array}{ccc} & & l(MR(1),MR(2),\cdots ,MR(D)|S(1),S(2),\cdots ,S(D),\alpha )\\ & = & \ln L(MR(1),MR(2),\cdots ,MR(D)|S(1),S(2),\cdots ,S(D),\alpha )\\ & = & -\frac{D}{2}\ln\left(2\pi \sigma^{2}\right)-\frac{1}{2\sigma^{2}}\sum_{k=1}^{D}v^{2}\left(k\right)+\ln h. \end{array}$$

To maximize the logarithm likelihood function l(MR(1), MR(2), ⋯, MR(D)|S(1), S(2), ⋯, S(D), α) in (17), we made its derivative equal to zero and obtained the solution

(18) $$\sigma^{2}=\frac{1}{D}\sum_{k=1}^{D}v^{2}\left(k\right).$$

Inserting (18) into (17) gives

(19) $$\begin{array}{ccc} & & l(MR(1),MR(2),\cdots ,MR(D)|S(1),S(2),\cdots ,S(D),\alpha )\\ & = & -\frac{D}{2}\ln\left(2\pi \right)-\frac{D}{2}+\ln h-\frac{D}{2}\ln\frac{1}{D}\sum_{k=1}^{D}v^{2}\left(k\right)\\ & = & \mathrm{const}-\frac{D}{2}\ln\frac{1}{D}\sum_{k=1}^{D}v^{2}\left(k\right). \end{array}$$

In view of (19) and (5), the MLFF J(α) is defined as

$$\begin{array}{ccc} J(\alpha ) & = & \frac{1}{D}\sum_{k=1}^{D}{\left[MR\left(k\right)-\varphi^{T}\left(k\right)\alpha \right]}^{2}. \end{array}$$

Therefore, the logarithm likelihood function l(MR(1), MR(2), ⋯, MR(D)|S(1), S(2), ⋯, S(D), α) in (19) could be rewritten as

(20) $$l\left(MR\left(1\right),MR\left(2\right),\cdots ,MR\left(D\right)|S\left(1\right),S\left(2\right),\cdots ,S\left(D\right),\alpha \right)=\mathrm{const}-\frac{D}{2}\ln J\left(\alpha \right).$$

From (20), the maximum value of the logarithm likelihood function l(MR(1), MR(2), ⋯, MR(D)|S(1), S(2), ⋯, S(D), α) could be obtained by minimizing the MLFF,

(21) $$J\left(\alpha \right)=\frac{1}{D}\sum_{k=1}^{D}{\left[MR\left(k\right)-\varphi^{T}\left(k\right)\alpha \right]}^{2}=\min.$$

### 4.5. Selection Process

At this stage, the fitness of the crossover vector ${\hat{\lambda }}_{p}^{gen+1}$ and the individual ${\hat{\alpha }}_{p}^{gen}$ are assessed by calculating their MLFFs $J({\hat{\lambda }}_{p}^{gen+1})$ and $J({\hat{\alpha }}_{p}^{gen})$ and the greedy selection strategy is adopted to determine whether the crossover vector ${\hat{\lambda }}_{p}^{gen+1}$ or the individual ${\hat{\alpha }}_{p}^{gen}$ remains in the population. From (21), it could be seen that the smaller value of the MLFF means better fitness. The selection process is described by the following equations:

(22) $$\begin{array}{ccc} J({\hat{\lambda }}_{p}^{gen+1}) & = & \frac{1}{D}\sum_{k=1}^{D}{\left[MR\left(k\right)-\varphi^{T}\left(k\right){\hat{\lambda }}_{p}^{gen+1}\right]}^{2}, \end{array}$$

(23) $$\begin{array}{ccc} J({\hat{\alpha }}_{p}^{gen}) & = & \frac{1}{D}\sum_{k=1}^{D}{\left[MR\left(k\right)-\varphi^{T}\left(k\right){\hat{\alpha }}_{p}^{gen}\right]}^{2}, \end{array}$$

(24) $$\begin{array}{ccc} {\hat{\alpha }}_{p}^{gen+1} & = & \left\{\begin{array}{cc} {\hat{\lambda }}_{p}^{gen+1}, & \mathrm{if}\;J\left({\hat{\lambda }}_{p}^{gen+1}\right)<J\left({\hat{\alpha }}_{p}^{gen}\right)\\ {\hat{\alpha }}_{p}^{gen}, & \mathrm{if}\;J\left({\hat{\lambda }}_{p}^{gen+1}\right)⩾J\left({\hat{\alpha }}_{p}^{gen}\right) \end{array}\right.. \end{array}$$

According to (24), if the MLFF of the crossover vector ${\hat{\lambda }}_{p}^{gen+1}$ is smaller, the individual ${\hat{\alpha }}_{p}^{gen+1}$ at the generation gen+1 would take the place of the crossover vector ${\hat{\lambda }}_{p}^{gen+1}$; otherwise, the individual ${\hat{\alpha }}_{p}^{gen}$ would remain in the population until generation gen+1.

Afterward, we could find the best individual ${\hat{\alpha }}_{best}^{gen+1}$ at the generation gen+1 in the population ${\hat{\Lambda }}^{gen+1}={\left[{\hat{\alpha }}_{1}^{gen+1},{\hat{\alpha }}_{2}^{gen+1},\cdots ,{\hat{\alpha }}_{P}^{gen+1}\right]}^{T}$ through the following equation:

(25) $$\begin{array}{ccc} {\hat{\alpha }}_{best}^{gen+1} & = & \underset{{\hat{\alpha }}_{p}^{gen+1}}{\arg\min}J\left({\hat{\alpha }}_{p}^{gen+1}\right)\\ & = & \underset{{\hat{\alpha }}_{p}^{gen+1}}{\arg\min}\frac{1}{D}\sum_{k=1}^{D}{\left[MR\left(k\right)-\varphi^{T}\left(k\right){\hat{\alpha }}_{p}^{gen+1}\right]}^{2},\;p=1,2,\cdots ,P. \end{array}$$

When $gen<gen_{\max}-1$, let gen increase by 1 and repeat the mutation process, the crossover process, and the selection process to update the individual ${\hat{\alpha }}_{p}^{gen+1}$ in the population ${\hat{\Lambda }}^{gen+1}$. When $gen=gen_{\max}-1$, the best individual ${\hat{\alpha }}_{best}^{gen_{\max}}$ is the final estimate of the parameter vector α.

**Figure 3:**
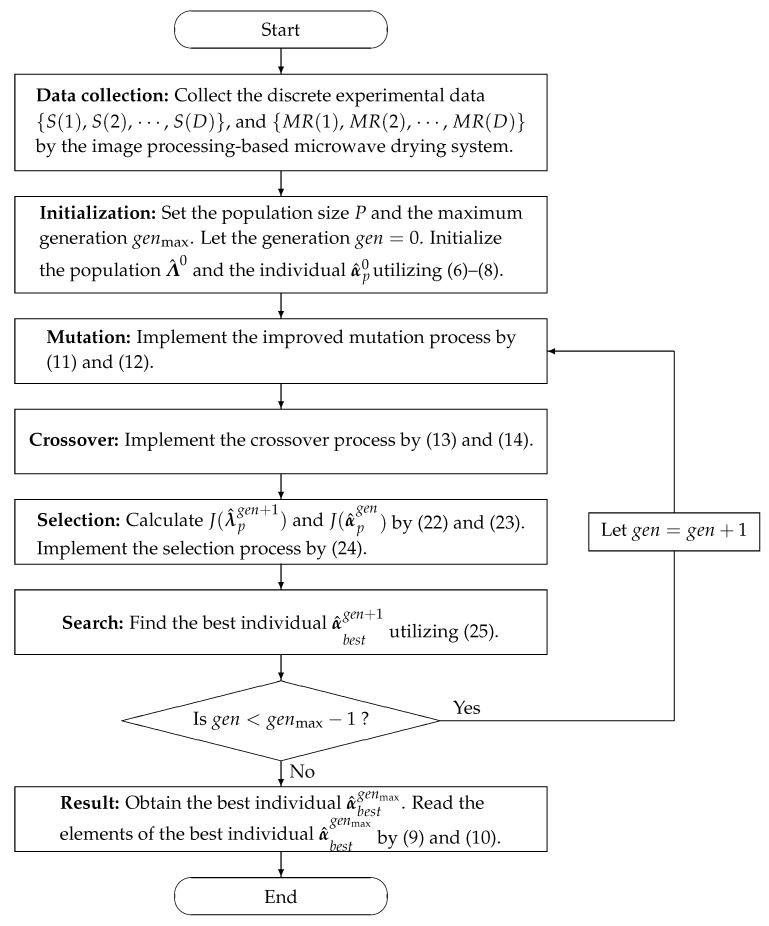
The flowchart of the MLFF-PE algorithm.

The flowchart utilizing the MLFF-PE algorithm in (6)–(14) and (22)–(25) to estimate the parameter vector α of the model in (5) is displayed in Figure 3.

From Figure 3, it can be seen that there are seven main steps of the MLFF-PE algorithm. In the stage of data collection, the discrete experimental data {S(1), S(2), ⋯, S(D)} and {MR(1), MR(2), ⋯, MR(D)} are collected by the image processing-based microwave drying system. In the stage of initialization, the initial values of the MLFF-PE algorithm are given. In the stage of mutation, the mutant vector ${\hat{\beta }}_{p}^{gen+1}$ is produced for each candidate solution ${\hat{\alpha }}_{p}^{gen}$. In the stage of crossover, the crossover vector ${\hat{\lambda }}_{p}^{gen+1}$ is constructed by mixing some elements in the mutant vector ${\hat{\beta }}_{p}^{gen+1}$ and some elements in the individual ${\hat{\alpha }}_{p}^{gen}$. In the stage of selection, the MLFFs of the crossover vector ${\hat{\lambda }}_{p}^{gen+1}$ and the individual ${\hat{\alpha }}_{p}^{gen}$ are calculated and compared to determine whether the crossover vector ${\hat{\lambda }}_{p}^{gen+1}$ or the individual ${\hat{\alpha }}_{p}^{gen}$ remained in the population. In the stage of the search, the best individual ${\hat{\alpha }}_{best}^{gen+1}$ is chosen from the population ${\hat{\Lambda }}^{gen+1}$ by comparing the MLFFs of all individuals ${\hat{\alpha }}_{1}^{gen+1}$, ${\hat{\alpha }}_{2}^{gen+1}$, ⋯, ${\hat{\alpha }}_{P}^{gen+1}$ at the generation gen+1. Finally, if $gen<gen_{\max}-1$, let gen=gen+1, and repeat the iteration process; otherwise, obtain the best individual ${\hat{\alpha }}_{best}^{gen_{\max}}$.

The MLFF-PE algorithm is a population-based estimation algorithm. That is to say, there are *P* parameter estimation vectors ${\hat{\alpha }}_{1}^{gen_{\max}}$, ${\hat{\alpha }}_{2}^{gen_{\max}}$, ⋯,${\hat{\alpha }}_{P}^{gen_{\max}}$ at $gen_{\max}$. Therefore, by calculating the MLFFs of the above parameter estimation vectors, we can choose one parameter estimation vector ${\hat{\alpha }}_{best}^{gen_{\max}}$ with the best MLFF as the final estimate of the parameter vector α. Therefore, the final parameter estimation vector ${\hat{\alpha }}_{best}^{gen_{\max}}$ is called the best parameter estimate.

## 5. Modeling and Prediction

In model fitting and prediction, we used two different batches of discrete experimental data—the shrinkage of the samples {S(1), S(2), ⋯, S(D)} and the moisture ratio of the samples {MR(1), MR(2), ⋯, MR(D)} during the drying process of Hami melon slices sampled by the developed image processing-based microwave drying system.

### 5.1. Model Fitting

The different orders n=1, n=2, and n=3 are set for the model in (5) and the MLFF-PE algorithm (the population size P=30 and the maximum generation $gen_{\max}=20$) are exploited to produce the best parameter estimates. To evaluate the model-fitting ability of the MLFF-PE algorithm and choose the optimal order, the coefficient of determination $R^{2}$ is computed by

$$R^{2}=1-\frac{\sum_{k=1}^{D}{\left[{\hat{MR}}_{est}\left(k\right)-MR\left(k\right)\right]}^{2}}{\sum_{k=1}^{D}{\left[MR\left(k\right)-\bar{MR}\right]}^{2}}=1-\frac{\sum_{k=1}^{D}{\left[\varphi^{T}\left(k\right){\hat{\alpha }}_{best}^{gen_{\max}}-MR\left(k\right)\right]}^{2}}{\sum_{k=1}^{D}{\left[MR\left(k\right)-\bar{MR}\right]}^{2}},$$

where ${\hat{MR}}_{est}\left(k\right)$ is the estimated moisture ratio, MR(k) is the experimental moisture ratio, and $\bar{MR}$ is the average value of the experimental moisture ratios. For the same order (i.e., the number of parameters to be estimated), the larger the coefficient of determination $R^{2}$ is, the better the model-fitting ability of the MLFF-PE algorithm. However, the model orders also affect the value of $R^{2}$. When the model orders are different, $R^{2}$ cannot be used to evaluate the model-fitting ability. Hence, the adjusted coefficient of determination $adjR^{2}$ is introduced:

$$adjR^{2}=1-\frac{\left(1-R^{2}\right)\left(D-1\right)}{D-(n+1)},$$

which considers the number of discrete experimental data *D* and the number of parameters to be estimated n+1. The larger adjusted coefficient of determination $adjR^{2}$ indicates the better model-fitting ability of the MLFF-PE algorithm.

The root-mean-square error (RMSE) is also used to evaluate the model-fitting ability and it is defined as follows:

$$\;\mathrm{RMSE}={\left\{\frac{1}{D}\sum_{k=1}^{D}{\left[{\hat{MR}}_{est}\left(k\right)-MR\left(k\right)\right]}^{2}\right\}}^{\frac{1}{2}}={\left\{\frac{1}{D}\sum_{k=1}^{D}{\left[\varphi^{T}\left(k\right){\hat{\alpha }}_{best}^{gen_{\max}}-MR\left(k\right)\right]}^{2}\right\}}^{\frac{1}{2}}.$$

The smaller the RMSE is, the better the model-fitting ability of the MLFF-PE algorithm.

**Figure 4:**
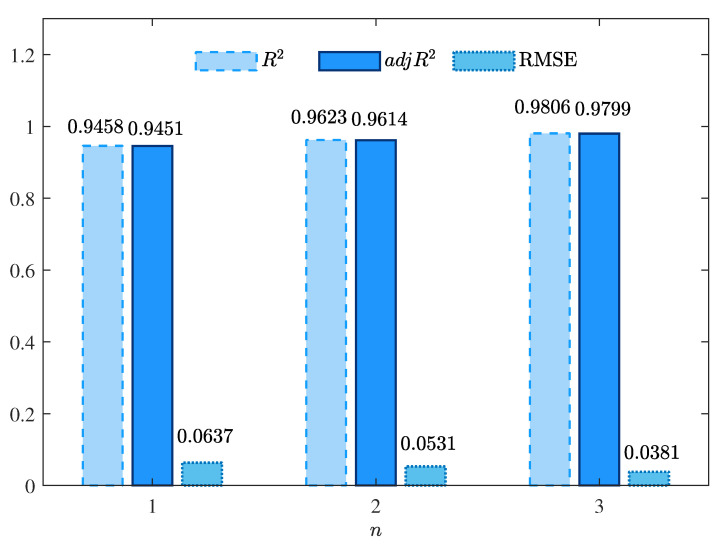
The values of $R^{2}$, $adjR^{2}$, and RMSEs given by the MLFF-PE algorithm for different orders *n*.

**Figure 5:**
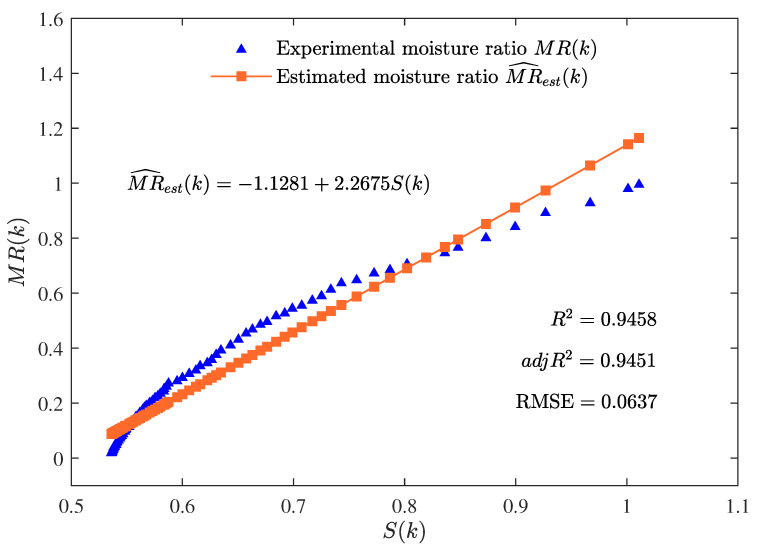
The estimated moisture ratio ${\hat{MR}}_{est}\left(k\right)$ versus the shrinkage S(k) for n=1.

**Figure 6:**
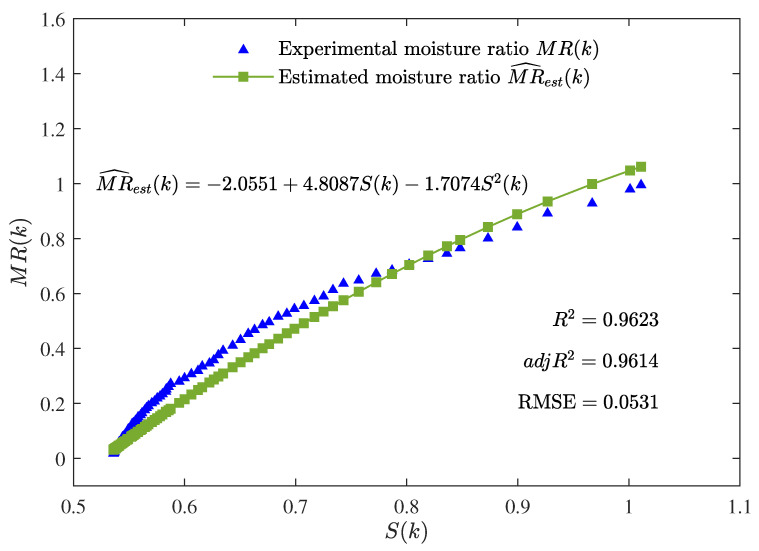
The estimated moisture ratio ${\hat{MR}}_{est}\left(k\right)$ versus the shrinkage S(k) for n=2.

**Figure 7:**
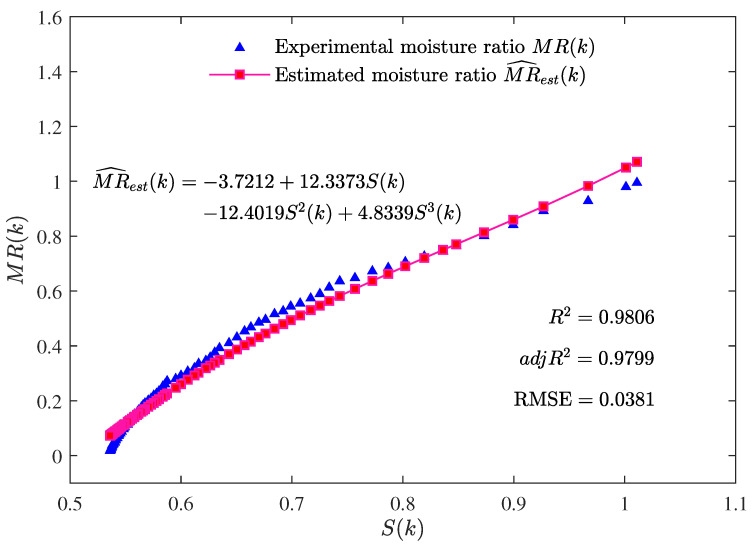
The estimated moisture ratio ${\hat{MR}}_{est}\left(k\right)$ versus the shrinkage S(k) for n=3.

The best parameter estimates given by the MLFF-PE algorithm for n=1, n=2, and n=3 are summarized in Table 1. Additionally, the values of $R^{2}$, $adjR^{2}$, and RMSE given by the MLFF-PE algorithm for n=1, n=2, and n=3 are shown in Table 1 and Figure 4. The curves of the estimated moisture ratio ${\hat{MR}}_{est}\left(k\right)$ and the experimental moisture ratio MR(k) versus the shrinkage S(k) for n=1, n=2, and n=3 are depicted in Figure 5, Figure 6 and Figure 7.

### 5.2. Prediction

In Table 1, the best parameter estimate ${\hat{\alpha }}_{best}^{gen_{\max}}$ given by the MLFF-PE algorithm for n=3 is used to compute the predicted moisture ratio ${\hat{MR}}_{pre}\left(k\right)$:

$${\hat{MR}}_{pre}\left(k\right)=\varphi^{T}\left(k\right){\hat{\alpha }}_{best}^{gen_{\max}}=-3.7212+12.3373S\left(k\right)-12.4019S^{2}\left(k\right)+4.8339S^{3}\left(k\right).$$

The coefficient of determination $R^{2}$ of the predicted model is computed by

$$R^{2}=1-\frac{\sum_{k=1}^{D}{\left[{\hat{MR}}_{pre}\left(k\right)-MR\left(k\right)\right]}^{2}}{\sum_{k=1}^{D}{\left[MR\left(k\right)-\bar{MR}\right]}^{2}}=1-\frac{\sum_{k=1}^{D}{\left[\varphi^{T}\left(k\right){\hat{\alpha }}_{best}^{gen_{\max}}-MR\left(k\right)\right]}^{2}}{\sum_{k=1}^{D}{\left[MR\left(k\right)-\bar{MR}\right]}^{2}}=0.9782,$$

the adjusted coefficient of determination $adjR^{2}$ of the predicted model is computed by

$$adjR^{2}=1-\frac{\left(1-R^{2}\right)\left(D-1\right)}{D-(n+1)}=0.9774,$$

and the RMSE of the predicted model is computed by

$$\;\mathrm{RMSE}={\left\{\frac{1}{D}\sum_{k=1}^{D}{\left[{\hat{MR}}_{pre}\left(k\right)-MR\left(k\right)\right]}^{2}\right\}}^{\frac{1}{2}}={\left\{\frac{1}{D}\sum_{k=1}^{D}{\left[\varphi^{T}\left(k\right){\hat{\alpha }}_{best}^{gen_{\max}}-MR\left(k\right)\right]}^{2}\right\}}^{\frac{1}{2}}=0.0404.$$

**Figure 8:**
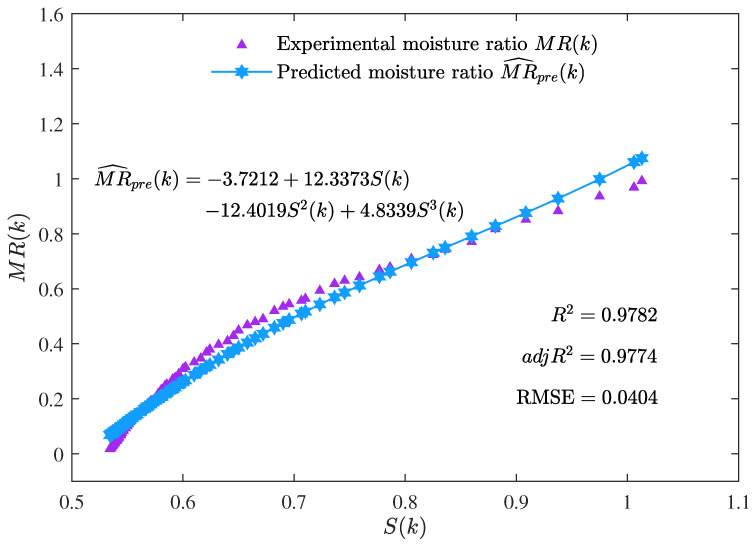
The predicted moisture ratio ${\hat{MR}}_{pre}\left(k\right)$ versus the shrinkage S(k).

**Figure 9:**
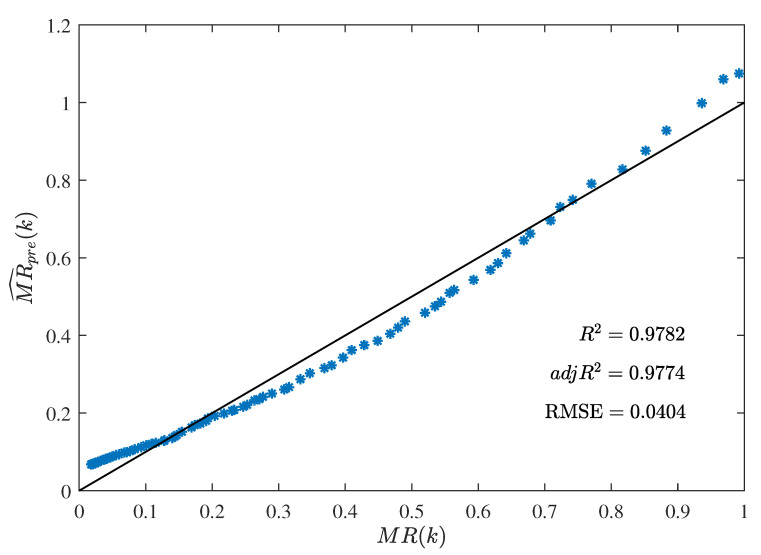
The comparison between the predicted moisture ratio ${\hat{MR}}_{pre}\left(k\right)$ and the experimental moisture ratio MR(k).

The curve of the predicted moisture ratio ${\hat{MR}}_{pre}\left(k\right)$ versus the shrinkage S(k) is illustrated in Figure 8. The comparison between the predicted moisture ratio ${\hat{MR}}_{pre}\left(k\right)$ and the experimental moisture ratio MR(k) is illustrated in Figure 9.

### 5.3. Results and Discussion

The parameter estimates given by the MLFF-PE algorithm for n=1, n=2, and n=3 are summarized in Table 1 and the values of $R^{2}$, $adjR^{2}$, and RMSE given by the MLFF-PE algorithm for n=1, n=2, and n=3 are shown in Table 1 and Figure 4. The curves of the estimated moisture ratio ${\hat{MR}}_{est}\left(k\right)$ and the experimental moisture ratio MR(k) versus the shrinkage S(k) for n=1, n=2, and n=3 are displayed in Figure 5, Figure 6 and Figure 7. It could be deduced from Table 1 and Figure 4, Figure 5, Figure 6 and Figure 7 that the MLFF-PE algorithm is effective for model fitting because the estimated moisture ratio ${\hat{MR}}_{est}\left(k\right)$ is close to the experimental moisture ratio MR(k). Meanwhile, n=3 is selected as the optimal order because the adjusted coefficient of determination $adjR^{2}=0.9799$ for n=3 is larger than $adjR^{2}=0.9451$ for n=1 and $adjR^{2}=0.9614$ for n=2, and the RMSE=0.0381 for n=3 is smaller than RMSE=0.0637 for n=1 and RMSE=0.0531 for n=2. A previous study [39] used the linear model and the ANN model to estimate the moisture ratio versus the shrinkage of potato slices during the drying process. For the linear model, $R^{2}=0.9310$ and RMSE=0.1398. For the ANN model, $R^{2}=0.9575$ and RMSE=0.1015. In this paper, for the linear model (n=1), $R^{2}=0.9458$ and RMSE=0.0637. For the two-order polynomial model (n=2), $R^{2}=0.9623$ and RMSE=0.0531. For the three-order polynomial model (n=3), $R^{2}=0.9806$ and RMSE=0.0381. Therefore, the estimated moisture ratio model ${\hat{MR}}_{est}\left(k\right)$ given by the proposed MLFF-PE algorithm is more accurate.

The curve of the predicted moisture ratio ${\hat{MR}}_{pre}\left(k\right)$ versus the shrinkage S(k) is illustrated in Figure 8. The comparison between the predicted moisture ratio ${\hat{MR}}_{pre}\left(k\right)$ and the experimental moisture ratio MR(k) is illustrated in Figure 9. It could be seen from Figure 8 and Figure 9 that the predicted moisture ratio ${\hat{MR}}_{pre}\left(k\right)$ is very close to the experimental moisture ratio MR(k). In other words, the estimated moisture ratio model given by the MLFF-PE algorithm performed well in prediction with the adjusted coefficient of determination $adjR^{2}=0.9774$ and the root-mean-square error RMSE=0.0404. A previous study [39] used the linear model and the ANN model to predict the moisture ratio versus the shrinkage of potato slices during the drying process. For the linear model, $R^{2}=0.9239$ and RMSE=0.1340. For the ANN model, $R^{2}=0.9687$ and RMSE=0.0966. In this paper, for the three-order polynomial model (n=3), $R^{2}=9782$ and RMSE=0.0404. Therefore, the predicted moisture ratio model ${\hat{MR}}_{est}\left(k\right)$ given by the proposed MLFF-PE algorithm is more accurate.

## 6. Conclusions

In this paper, an experimental system (that included an adjustable-power microwave drying unit and an image-processing unit) was built for the Hami melon drying process. The mathematical model of the moisture ratio with regard to the shrinkage of the drying process of Hami melon slices was built according to the Weierstrass approximation theorem, and the MLFF-PE algorithm was developed to fit the moisture ratio model and predict the moisture ratio. The results revealed that the presented MLFF-PE algorithm was effective for model fitting and prediction. Compared with the previous study, both the estimated and predicted moisture ratio models given by the proposed MLFF-PE algorithm were more accurate. In future work, we will study the improved algorithms to obtain better estimation and prediction results of the moisture ratio models during food microwave drying processes.

## Figures and Tables

**Table 1:** The best parameter estimates given by the MLFF-PE algorithm for different orders *n*.

*n*	${\hat{\alpha }}_{0,\mathrm{best}}^{{\mathrm{gen}}_{\max}}$	${\hat{\alpha }}_{1,\mathrm{best}}^{{\mathrm{gen}}_{\max}}$	${\hat{\alpha }}_{2,\mathrm{best}}^{{\mathrm{gen}}_{\max}}$	${\hat{\alpha }}_{3,\mathrm{best}}^{{\mathrm{gen}}_{\max}}$	$R^{2}$	${\mathrm{adjR}}^{2}$	RMSE
1	−1.1281	2.2675	–	–	0.9458	0.9451	0.0637
2	−2.0551	4.8087	−1.7074	–	0.9623	0.9614	0.0531
3	−3.7212	12.3373	−12.4019	4.8339	0.9806	0.9799	0.0381

## Data Availability

The data in this research are available upon request from the corresponding author.
